# Activity of *Mentha piperita* L. Ethanol Extract against Acetic Acid Bacteria *Asaia* spp.

**DOI:** 10.3390/foods7100171

**Published:** 2018-10-18

**Authors:** Hubert Antolak, Agata Czyżowska, Dorota Kręgiel

**Affiliations:** Institute of Fermentation Technology and Microbiology, Lodz University of Technology, Wolczanska 171/173, 90-924 Lodz, Poland; agata.czyzowska@p.lodz.pl (A.C.); dorota.kregiel@p.lodz.pl (D.K.)

**Keywords:** *Asaia* spp., *Mentha piperita* L., antibacterial effect, antiadhesion effect, functional beverages

## Abstract

Acetic acid bacteria belonging to the genus *Asaia* spp. are relatively new microbial contaminants in the beverage industry. These bacteria cause organoleptic changes such as increased turbidity, haziness and sour odor. In addition, they are able to form biofilms on the inner parts of production lines, and finally they can cause secondary contamination of final products. For this reason, new methods using effective and safe preservatives are being developed to improve microbial stability of soft beverages. The aim of the research was to investigate the effects of *Mentha piperita* L. ethanol extract against *Asaia* spp. biofilm formation. The bacterial adhesion was evaluated by a plate count method and luminometry, as well as fluorescence microscopy. The polyphenolic profile of the mint extract was determined on the basis of high-performance liquid chromatography (HPLC). The obtained microbiological results indicate bacteriostatic effect of mint extract at 10% (*v*/*v*) concentration. The plant extract also reduces the number of adhered bacterial cells on polystyrene surface.

## 1. Introduction

Soft drinks constitute a diverse group of products. Their classification depends on the basis of sugar or sweetener content, fruit juice and flavor addition, carbonation level, as well as their functionality. Functional beverages, except water, sweeteners, foaming agents, preservatives, aromas and colorants, contain bioactive substances such as vitamins, minerals, amino acids and proteins, dietary fiber, caffeine or taurine [1]. In addition to their basic function—body hydration—functional beverages show positive effects on consumer well-being and health. On the other hand, the addition of specific functional additives may lead to the microbial instability of final products, providing additional nutrients for spoilage microorganisms. In addition, the low pH level of functional drinks reaching value below 4, along with the addition of conventional preservatives (sodium benzoate, potassium sorbate, dimethyl dicarbonate) are not usually sufficient to ensure microbial stability of final products. Soft drinks can be a favorable environment for the growth of yeasts, molds and acidophilic bacteria. Acetic acid bacteria belonging to the genera *Acetobacter*, *Gluconobacter* are often isolated from non-alcoholic drinks [2,3]. However, in recent years, functional drinks have often been contaminated by other acetic acid bacteria belonging to the genus *Asaia* [4,5,6,7]. Our long cooperation with Polish drinks factories shows that *Asaia* spp. growth occurs despite the low pH of the beverages and the addition of chemical preservatives [7]. As a result, manufacturers take steps aimed at preventing the appearance of defective products on the market. Therefore, our study is the consequence of cooperation with the companies interested in unknown spoilage microorganisms, which are difficult to detect and identify.

The growth of *Asaia* spp. in soft drinks results in formation of flocs and sediments, turbidity or color changes [8,9]. What is more, these bacteria show adhesion ability to various abiotic materials commonly used as packaging or installation pipes and accessories [10,11,12,13]. It is worth noting that biofilms formed on the inner surfaces of production lines can cause secondary contamination of final products.

Many functional drinks have been developed to provide specific medical or health benefits, such as promoting heart health, improving immunity and digestion, and helping to boost energy. Due to the trend of healthy food, the increasing interest of natural, plant-origin additives has been observed [10,13,14]. One of the most popular families of medicinal plants is Lamiaceae. These plants are used in traditional and modern medicine, as well as in the food industry. Representatives of this family are commonly found in Europe, Asia, North America and North Africa. The fresh and dried parts of *Mentha* spp., as well as their extracts, are used in aromatherapy, pharmacology and nutrition. In the food industry, the essential oils and extracts of mint are used as flavoring agents in various products, including: cheese, chocolate, soft drinks, jellies, syrups, candies and chewing gums [15]. It is worth noting that mint phytochemicals can be successfully used as natural food preservatives to prevent the growth of spoilage microorganisms [15]. The mint extracts can be used as additives to prolong the shelf-life of products, eliminating or reducing the use of synthetic preservatives and flavorings. In our work, we used ethanol extract of mint as a natural antibacterial agent against spoilage acetic acid bacteria *Asaia* spp. isolated from commercial soft drinks in Poland.

## 2. Materials and Methods

### 2.1. Materials

#### 2.1.1. Plant Extract

*Mentha piperita* L. were harvested in the Lodz region (Poland) in May 2017. After harvesting, plants were washed and gently dried on paper towels and then tied in loose bunches. To protect them from dust and contamination, they were put into paper bags with small punch holes which ensured good ventilation and air circulation. Drying was carried out in a dark room at 30 °C for 30 days. Then dried leaves were crushed using a laboratory mortar and 50 g were then placed in 500 mL dark glass bottle filled with 250 mL of 10% (*v*/*v*) ethanol. Then bottles were stored at room temperature for one month with agitation [16]. Subsequently, the macerated leaves were centrifuged at 6500 rpm for 10 min. at 15 °C (Eppendorf, Hamburg, Germany). Such prepared plant extract was added to the culture media to a final concentration of 10% (*v*/*v*), while the final concentration of ethanol in a culture medium was approximately 1% (*v*/*v*). The liquid culture media with plant extracts were sterilized using microfiltration with 0.45-μm-pore-size membranes (Merck Millipore, Darmstadt, Germany) [16].

#### 2.1.2. Bacterial Strains

Antimicrobial and antiadhesive activities of 10% (*v*/*v*) mint extract (ME) were evaluated against 6 bacterial strains of *Asaia* spp. characterized by strong adhesive abilities: *As. bogorensis* ISD1 (GenBank KP234014), *As. bogorensis* ISD2 (GenBank KP234015), *As. bogorensis* FFMW (GenBank KC756841), *As. lannensis* IFCW (GenBank KP234012), *As. lannensis* FMW1 (GenBank HQ917850), and *As. lannensis* W4 (GenBank MF777040). The pure cultures of bacterial strains were deposited in the Pure Culture Collection of Industrial Microorganisms LOCK 105, at the Institute of Fermentation Technology and Microbiology, Lodz University of Technology (Lodz, Poland). The bacterial strains were stored in liquid GC medium at +4 °C. Before each experiment, strains were activated by transfer into new liquid GC media and incubation at 25 °C for 4 days.

### 2.2. Methods

#### 2.2.1. Antibacterial Activity of Mint Extract

The effect of *M. piperita* L. ethanol extract on the growth of *Asaia* spp. was investigated in liquid minimal medium (2% (*w*/*v*) glucose, 0.3% (*w*/*v*) (NH_4_)_3_PO_4_, 0.3% (*w*/*v*) KH_2_PO_4_, 0.3% (*w*/*v*) MgSO_4_·7H_2_O, 0.05% (*w*/*v*) yeast extract) using a modified standard method (according to EN 1040:2005) with 96-well plates. For this purpose, 100 µL of 25% (*v*/*v*) *M. piperita* L. ethanol extract was added to 150 µL of minimal medium and mixed. Next, each microplate cell was inoculated with 50 µL of the standardized bacterial suspension. The final concentration of mint extract was 10% (*v*/*v*) and concentration of bacterial cells was approximately 10^5^–10^6^ cells per mL. The control sample was inoculated minimal medium without extract. Multiplates were incubated using a MULTISKAN GO spectrophotometer (Thermo Fisher Scientific, Waltham, MA, USA) at 25 °C for 40 h. Measurements of absorbance were carried out automatically every 1 h at a wavelength of 540 nm. The results are shown as the difference in absorbance (ΔAbs) after the measurement carried out for each hour (Abs_t_) and absorbance of medium at the beginning of the experiment (Abs_0_).

#### 2.2.2. Bacterial Adhesion

For adhesion studies, sterile polystyrene (PS) (Coveris Rigid Poland, Skierniewice, Poland) rectangles measuring 76 × 26 mm were used. This material is certified by the Polish National Institute of Public Health and is approved for contact with food.

The adhesion and biofilm formation by bacterial strains was evaluated in liquid minimal medium (2% (*w*/*v*) sucrose, 0.3% (*w*/*v*) (NH_4_)_3_PO_4_, 0.3% (*w*/*v*) KH_2_PO_4_, 0.3% (*w*/*v*) MgSO_4_·7H_2_O, 0.05% (*w*/*v*) yeast extract) with mint extract to obtain a final 10% (*v*/*v*) concentration. 20 mL of sterile medium was poured aseptically into 25 mL Erlenmeyer flasks covered with a textile cloth in order to ensure aerobic conditions. Sterile rectangular carriers were placed vertically into the liquid culture medium in such a way that half of the carrier was immersed in the medium. The culture media were inoculated by standardized bacterial suspension, and final concentration of bacterial cells was approximately 10^6^ cells per mL at the beginning of the experiment. The cultures were incubated at 25 °C for 6 days on a laboratory rotary shaker at 130 rpm.

The adhesion analysis was performed by the plate count method and luminometric measurements described by Kregiel (2013) [12]. To determine the number of the bacterial cells attached to tested surfaces, the carriers were removed from the media and swabbed with a sterile contact swabs. Subsequently, the removed bacterial biofilm was placed in a saline solution with 0.1% (*w*/*v*) Tween 80, vortexed and the appropriate dilutions were prepared. Then dilutions were transferred onto GC agar medium (2% (*w*/*v*) glucose, 0.3% (*w*/*v*) yeast extract, 0.3% (*w*/*v*) peptone, 0.7% (*w*/*v*) CaCO_3_, 2% (*w*/*v*) agar) and incubated for 96 h at 25 °C. After incubation, the colonies of *Asaia* spp. were counted and the results expressed as colony forming units per square centimeter (CFU/cm^2^) were determined. Additionally, the number of bacterial cells in the culture medium was evaluated, and the results expressed as colony forming units per milliliter of medium (CFU/mL). From the obtained values, the relative adhesion coefficient A (%) was calculated using the formula A (%) = (N_a_/N_p_) × 100%, where N_a_ is the number of attached cells to a carrier, and N_p_ is the number of planktonic cells in the culture medium. For luminometric analysis, the carriers were removed from the media, washed with sterile distilled water and swabbed with pens for ATP (adenosine triphosphate) sampling. Measurements were made using a HY-LiTE^®^2 luminometer (Merck-Millipore, Darmstadt, Germany). The results are expressed as Relative Light Units per square centimeter (RLU/cm^2^) of carrier.

#### 2.2.3. Fluorescence Microscopy

Visualization of bacterial cells in biofilms was performed by staining using a LIVE/DEAD^™^ BacLight^™^ Bacterial Viability Kit (Thermo Fisher Scientific, Waltham, MA, USA) in accordance with the manufacturer’s procedure. Biofilms were gently washed with phosphate buffered saline (PBS) solution, and then the entire surface was covered with a staining solution. The samples were incubated in darkness for 20 min at 25 °C. Images were taken using fluorescence microscope OLYMPUS BX53 equipped with filters with excitation wavelength ranging from 470 nm to 630 nm, and a high-resolution digital color camera (Olympus, Tokyo, Japan) [14].

#### 2.2.4. Chemical Constituents Analysis

The phenolic compounds contained in the tested extract were characterized using high-performance liquid chromatography (HPLC) with a diode array detector (DAD) (Finnigan Surveyor-PDA Plus detector, Thermo Fisher Scientific, Waltham, MA, USA) and ChromQuest 5.0 chromatography software (Thermo Fisher Scientific). Separation was achieved on a Lichrospher RP 18–5 (250 mm by 4.6 mm, 5 µm packing; Hichrom, Reading, UK). The elution conditions were as follows: flow rate of 0.8 mL/min; oven temperature of 25 °C; solvent A (5% (*v*/*v*) formic acid), and solvent B (95% (*v*/*v*) acetonitrile). The injection volume was 50 µL. Detection was conducted at 280, 320, and 360 nm. The identification of compounds was carried out on the basis of the results for the standards. Gallic acid (Sigma-Aldrich, Saint Louis, MO, USA), neochlorogenic acid, chlorogenic acid (Sigma-Aldrich), epicatechin (Sigma-Aldrich), *p*-coumaric acid (Sigma-Aldrich), ferulic acid (Sigma-Aldrich), rosmarinic acid (Sigma-Aldrich), quercetin-3-rutinoside (Sigma-Aldrich), caffeic acid (Sigma-Aldrich), and quercetin (Sigma-Aldrich) were used as pure standard solutions. On the basis of results obtained for tested plant sample and pure standards, the individual components of mint extract were identified.

#### 2.2.5. Statistics

Means with standard deviations were calculated from the data obtained from three independent experiments. The mean values of adhesion results were compared using one-way repeated measures analysis of variance (ANOVA; OriginPro 8.1, OriginLab Corp., Northampton, MA, USA). Values with different letters are statistically different (*p* < 0.05). a—*p* ≥ 0.05; b—0.005 < *p* < 0.05; c—*p* < 0.005. The results were compared with data received for the control culture medium (without mint extract). Bacterial growth profiles were created in Plotly (https://plot.ly/#/)—a free web tool for data visualization.

## 3. Results and Discussion

### 3.1. Antibacterial Activity

**Figure 1:**
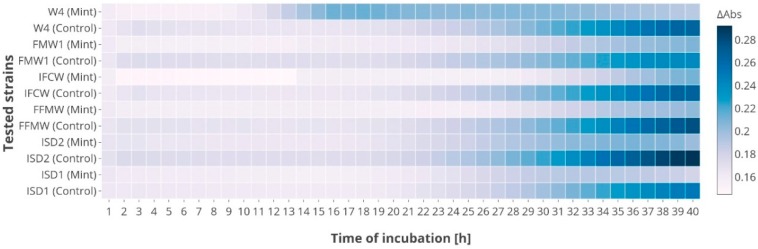
The growth of *As. bogorensis* (ISD1, ISD2, FFMW) and *As. lannensis* (IFCW, FMW1, W4) in the control culture medium and the medium with mint extract.

The biological activities of mint extract were evaluated against six strains of *Asaia* spp. under in vitro conditions. The tested ethanol extract showed weak bacteriostatic properties (Figure 1). The maximum value of absorbance after 40 h of cultivation in the control medium (minimal medium) was approximately 0.28. The best growth was obtained for *As. bogorensis* ISD2, while among *As. lannensis,* strain IFCW showed the best proliferation. However, in the medium containing 10% (*v*/*v*) extract of *M. piperita* L., bacterial growth was slightly inhibited, and the maximum value of absorbance was approximately 0.21. Antibacterial activity of mint extracts against Gram-positive and Gram-negative bacteria is well documented. Sujana et al., (2013) found that *M. piperita* L. leaf extract showed stronger activity against Gram-positive *Staphylococcus aureus (Staph. aureus)*, *Bacillus subtilis (B. subtilis)* than against Gram-negative *Escherichia coli (E. coli)* [17]. Antibacterial effect of peppermint water extract was also noted against *Pseudomonas aeruginosa (Ps. aeruginosa)* and *Serratia marcescens* [18]. The studies conducted by Laggoune et al., (2016) indicated that *E. coli* and *Proteus mirabilis* strains were sensitive to *Mentha spicata (M. spicata)* [19]. What is more, in the study of Dhiman et al. (2016), the authors tested the effect of acetone, methanol, ethanol, and water extracts of mint against spoiled juice-isolated microorganisms: *Bacillus cereus* and *Serratia* spp. [20]. Despite the fact that in the in vivo studies, the extract of mint showed a bacteriostatic effect, it should be remembered that this activity can be clearly different in a food industry environment. In general, biological activity can be influenced by food components (e.g., fats, carbohydrates, proteins, water, salt, preservatives), temperature, pH, water activity and packaging methods. Thus, to confirm the effectiveness of improving the stability and shelf-life of products through the growth inhibition of *Asaia* spp., it is necessary to conduct tests using the appropriate matrix.

### 3.2. Antiadhesive Activity

**Figure 2:**
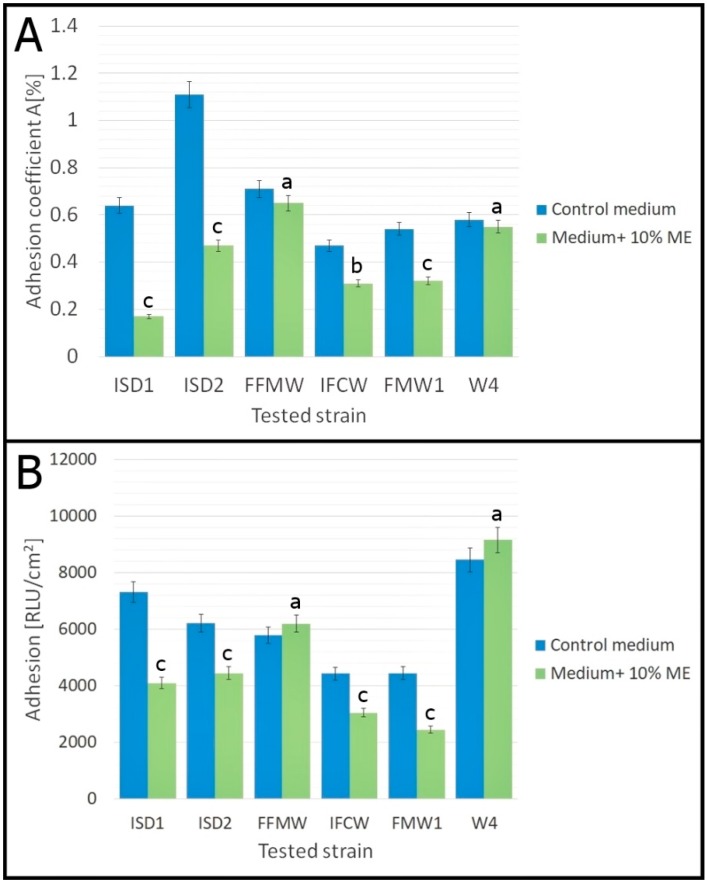
The effect of 10% (*v*/*v*) *M. piperita* L. mint extract (ME) on the adhesion of *As. bogorensis* (ISD1, ISD2, FFMW) and *As. lannensis* (IFCW, FMW1, W4). (**A**) Results of the plate count method. (**B**) Results of the luminometric method. Results obtained for control samples (without mint extract) were compared with results for samples with 10% (*v*/*v*) mint extract using one-way repeated measures analysis of variance (ANOVA). The values with different letters are statistically different (*p* < 0.05). a—*p* ≥ 0.05; b—0.005 < *p* < 0.05; c—*p* < 0.005. The results were compared with data received for the control medium.

Analysis of *Asaia* spp. adhesion to polystyrene surfaces was carried out in minimal medium with 10% (*v*/*v*) mint extract. The results of the plate count method and luminometry, expressed respectively as adhesion coefficient A (%) and adhesion (RLU/cm^2^), are presented in Figure 2. Both methods showed that *Asaia* spp. strains are characterized by strong adhesion properties. The relative coefficient A (%) for minimal medium ranged from 0.47% for *As. lannensis* IFCW to 1.11% for *As. lannensis* ISD2. The results of luminometry showed that adhesion ranged from 4430 RLU/cm^2^ for *As. lannensis* IFCW to 8450 RLU/cm^2^ for *As. bogorensis* W4. The results agree with those obtained by Kregiel and co-workers [11,12]. Our research confirmed that adhesion is a strain-dependent feature. According to the literature, adhesion of bacterial cells to different surfaces is usually correlated with the cell starvation. Biofilm formation occurs in rather poor environments, and in the case of *Asaia* spp., in the presence of sucrose as a carbon source. The knowledge of factors involved in biofilm formation by *Asaia* spp. is limited, but it has been shown that extracellular polymeric substances promote biofouling [12]. The physicochemical properties of abiotic materials play an important role in this phenomenon. It is known that adhesion to hydrophobic surfaces happens more intensively in comparison to hydrophilic surfaces with lower contact angles. Plastic materials, such as polystyrene, with a higher contact angle (87°) and low free energy (40 mN/m at 20 °C), are more hydrophobic than glass (contact angle 44° and surface free energy equal 70 mN/m at 20°). Thus, we may conclude that plastic packaging materials commonly used in beverage industry support bacterial adhesion and biofilm formation [21]. Therefore, novel effective antibiofilm strategies are urgently needed.

A great number of studies on antibiofilm strategies are focused on surface modification, and many approaches are based on active organosilanes [22,23,24] or nanostructured antibacterial multilayers [25,26]. Also, hygienic processes with the application of novel surfactants, stimulating biofilm eradication, have been used [27,28]. However, taking into account the dynamic development of the healthy food trend and the impact on the health and well-being of consumers, a modification of soft drink contents seems much more justified. An interesting alternative may be the addition of natural plant extracts and juices as a source of bioactive compounds to prevent microbial growth and adhesion.

The application of mint extract caused the reduction in cells adhesion of almost all *Asaia* spp. strains. The values of the relative adhesion coefficient A (%) show that 10% (*v*/*v*) mint extract inhibited adhesion of all strains of *As. bogorensis* and *As. lannensis* (Figure 2A). However, the reduction of coefficient A (%) for *As. bogorensis* FFMW and *As. lannensis* W4 was not statistically significant compared to the control medium (*p* ≥ 0.05). The highest decrease of A (%) values (73%), between adhesion in the medium with mint extract and adhesion in control medium, was noted for *As. bogorensis* ISD1. A slight decrease in the relative adhesion coefficient A (%) was noted for *As. bogorensis* FFMW and *As. lannensis* W4. On the other hand, the results obtained by the luminometric method were more divergent. According to the values of relative light units per square centimeter of the carrier [RLU/cm^2^], the antiadhesive effect of mint extract was significant for most of tested strains. Only for two strains—*As. bogorensis* FFMW and *As. lannensis* W4—was a slight increase in adhesion noted. However, these results were not statistically significant (*p* ≥ 0.05). The strongest antiadhesive activity was noted for *As. bogorensis* ISD1 and *As. lannensis* FMW1. For ISD1, the luminometric values were reduced from the 7320 RLU/cm^2^ to 4100 RLU/cm^2^ (decrease by 44%). Meanwhile, for FMW1, a reduction from 4450 RLU/cm^2^ to 2450 RLU/cm^2^ was noted.

**Figure 3:**
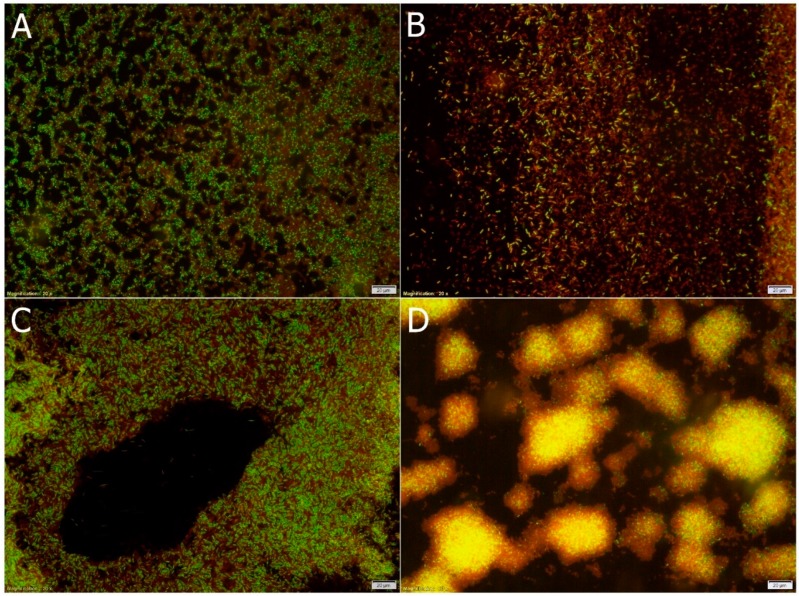
Microscopic observation of *As. bogorensis* ISD2 biofilms formed after 3-days-incubation (**A**,**B**) and 6-days-incubation (**C**,**D**) in minimal medium (**A**,**C**) and medium with 10% (*v*/*v*) *M. piperita* L. (**B**,**D**).

According to Figure 3, there are two potential mechanisms of inhibition of biofilm formation by mint extract. First of all, it may show antibacterial activity, which is evidenced by spectrophotometric results and fluorescence microscopic images. Figure 3B,D show biofilm formed after 3-day and 6-day incubation of *As. bogorensis* ISD2 in the medium with 10% (*v*/*v*) ME. In both cases, a significant proportion of dead (red) cells to live (green) was visible. What is more, in comparison to biofilms in the control sample (without mint extract), Figure 3A,C show a lower level of living cells. The bacterial viability kit used in our study was a mixture of SYTO^®^9 (green-fluorescent nucleic acid) stain and propidium iodide (red-fluorescent) stain. SYTO^®^9 used alone labels both bacteria with damaged membranes and those with intact membranes. In turn, propidium iodide exhibits activity only in relation to bacteria with damaged membranes, and at the same time, it causes SYTO^®^9 reduction. As a consequence, undamaged cells show green fluorescence while cells with damaged membranes are red [14]. On the other hand, the second mechanism may rely on direct anti-adhesion activities against *Asaia* spp. cells. By comparing the surfaces of biofilms, meaningfully smaller areas of biofilms were formed in the medium with the addition of the mint extract than in the case of in the control media. Literature data on antiadhesive and antibiofilm activities of mint extracts is limited. A few previous reports have shown that essential oils and extracts prepared from *M. piperita* inhibited biofilm formation by bacteria *Listeria monocytogenes*, *Pseudomonas aeruginosa*, as well as yeasts belonging to *Candida* species [29].

### 3.3. Chemical Composition

Plants of the genus *Mentha* show a great chemical variability. Reports on chemical composition of mint species showed that many factors interfere with bioactive compound composition, among them environmental (growth location, soil characteristics, moisture presence, temperature, etc.), phenological (phase of the plant collection), plant part used for extraction (flowers, stems, leaves, entire aerial parts or inflorescences), type of material (fresh or dry), and even methods used for chemical analysis. According to the literature, rosmarinic acid, luteolin-7-*O*-glucoside, salvianolic acid, eriocitrin and hesperidin have been found as the major non-volatile constituents in *Mentha species* [15]. As can be seen in Table 1, the mint extract used in the study was a source mainly of phenolic acids, such as gallic, chlorogenic, neochlorogenic, p-coumaric, ferulic as well as rosmarinic acids. In addition, the presence of epicatechin, quercetin-3-rutinoside and quercetin was also found.

The phenolic acid content, as well as the essential oils, in the mint extracts has an influence on the biological activities of this plant material. It has been documented that gallic and caffeic acids show activity against the Gram-negative *Klebsiella pneumoniae* and Gram-positive *Staphylococcus epidermidis*, as well as *Staph. aureus* [30]. It is well known that, along with phenolic acids, mint is a rich source of essential oils (EOs). It is believed that antimicrobial activity of the extract is the result of the synergistic actions of various bioactive compounds contained in the tested extract [18]. Dhifi et al., (2013) reported that *Mentha spicata* essential oil showed high activity against Gram-positive bacteria *Staph. epidermidis* and *Staph. aureus*, as well as Gram-negative cells of *Salmonella* spp. and *E. coli* [31]. Soković et al., (2010) found that *M. piperita* and *M. spicata* essential oils were more active than oils from sweet basil, lavender, sage or camomile against a wide spectrum of bacterial strains: *B. subtilis*, *E. coli* O157:H7, *Ps. aeruginosa*, *Proteus mirabilis*, *Salmonella* spp. and *Staph. aureus* [32]. They also noted that menthol was more active than other compounds extracted from tested plants: linalyl acetate, limonene, β-pinene, α-pinene, camphor, linalool and 1,8-cineole. In general, the better activity of both the extracts and essential oils of mint have been noted for Gram-positive bacteria. Presumably, the lower susceptibility of Gram-negative bacteria results inter alia from the presence of hydrophobic lipopolysaccharides in their outer membrane, which provides protection against different antimicrobial agents [33]. This structure prevents the depolarization, pores formation and increasing of membrane permeability [34]. The crucial factors affecting the antimicrobial activity include: the composition of used extract, the concentration of active substances as well as the type of tested microorganism. The composition of active substances may be determined by plant cultivation method, environmental conditions, time of plant harvesting, method of plant drying, and storage conditions, as well as extraction method [35].

## 4. Conclusions

The ethanol extract of *Mentha piperita* L. shows bacteriostatic properties and antiadhesive activity against *Asaia* spp. strains isolated from commercial soft drinks. Our study results suggested that *Mentha piperita* L. extract can be considered as a good source of natural compounds with significant antioxidant activity. The antiadhesive action of mint makes it an interesting component of soft drinks to counteract the formation of bacterial cell aggregates, flocks, haziness, biofilms and, finally, it will allow to find several applications for shelf-life extending of soft drinks.

## Figures and Tables

**Table 1:** Main phenolic compounds in mint extract identified by HPLC.

No.	Time	λ_max_ (nm)	Proposed Molecule
1	3.783	277	Gallic acid
2	11.073	324	Neochlorogenic acid
3	16.323	323	Chlorogenic acid
4	17.415	279	Epicatechin
5	20.868	308	*p*-Coumaric acid
6	23.787	322	Ferulic acid
7	30.947	290	Rosmarinic acid
8	32.133	318	Quercetin-3-rutinoside
9	33.993	322	Caffeic acid derivative
10	54.065	318	Quercetin

HPLC: high-performance liquid chromatography.
